# Generation and Characterization of Functional Cardiomyocytes Derived from Human T Cell-Derived Induced Pluripotent Stem Cells

**DOI:** 10.1371/journal.pone.0085645

**Published:** 2014-01-21

**Authors:** Tomohisa Seki, Shinsuke Yuasa, Dai Kusumoto, Akira Kunitomi, Yuki Saito, Shugo Tohyama, Kojiro Yae, Yoshikazu Kishino, Marina Okada, Hisayuki Hashimoto, Makoto Takei, Toru Egashira, Masaki Kodaira, Yusuke Kuroda, Atsushi Tanaka, Shinichiro Okata, Tomoyuki Suzuki, Mitsushige Murata, Jun Fujita, Keiichi Fukuda

**Affiliations:** 1 Department of Cardiology, Keio University School of Medicine, Shinjuku-ku, Tokyo, Japan; 2 Japan Society for the Promotion of Science, Chiyoda-ku, Tokyo, Japan; University of Kansas Medical Center, United States of America

## Abstract

Induced pluripotent stem cells (iPSCs) have been proposed as novel cell sources for genetic disease models and revolutionary clinical therapies. Accordingly, human iPSC-derived cardiomyocytes are potential cell sources for cardiomyocyte transplantation therapy. We previously developed a novel generation method for human peripheral T cell-derived iPSCs (TiPSCs) that uses a minimally invasive approach to obtain patient cells. However, it remained unknown whether TiPSCs with genomic rearrangements in the T cell receptor (TCR) gene could differentiate into functional cardiomyocyte in vitro. To address this issue, we investigated the morphology, gene expression pattern, and electrophysiological properties of TiPSC-derived cardiomyocytes differentiated by floating culture. RT-PCR analysis and immunohistochemistry showed that the TiPSC-derived cardiomyocytes properly express cardiomyocyte markers and ion channels, and show the typical cardiomyocyte morphology. Multiple electrode arrays with application of ion channel inhibitors also revealed normal electrophysiological responses in the TiPSC-derived cardiomyocytes in terms of beating rate and the field potential waveform. In this report, we showed that TiPSCs successfully differentiated into cardiomyocytes with morphology, gene expression patterns, and electrophysiological features typical of native cardiomyocytes. TiPSCs-derived cardiomyocytes obtained from patients by a minimally invasive technique could therefore become disease models for understanding the mechanisms of cardiac disease and cell sources for revolutionary cardiomyocyte therapies.

## Introduction

Severe heart failure is a progressive and treatment-resistant disease for which the only complete cure is heart transplantation [1]. In addition, many of the etiological problems leading to heart failure remain unsolved. Direct reprogramming of somatic cells to produce induced pluripotent stem cells (iPSCs) through the forced expression of several embryonic stem (ESC)-specific transcription factors is a prominent recent advance in stem cell biology [2], and human iPSCs hold great promise as new tools for investigating mechanism of disease and as cell sources for transplantation therapy [3]. Human iPSC-derived cardiomyocytes (iPSC-CMs) are thus potential future cell sources for novel cardiomyocyte transplantation therapies for severe heart failure [4], [5].

Successful differentiation of human iPSCs into cardiomyocytes was first reported in 2009 [6]. Subsequently, human iPSC-CMs have been developed as in vitro models for cardiac electrophysiological studies and drug screening [7]. Disease modeling of cardiac disease using patient-derived iPSC-CMs was also reported in the field of inherited arrhythmia diseases [8]–[12] and inherited cardiomyopathies [13]–[15]. Furthermore, iPSC-CMs have been engrafted successfully into the hearts of experimental animals [16] and used to improve cardiac function after ischemic cardiomyopathy in a porcine model [17]. Thus, research using iPSC-CM will be important in clarifying disease mechanisms and establishing novel therapies [18]–[20].

A wide range of donor cell types, gene-introducing vehicles, and combinations of reprogramming factors have been used for generating iPSCs [21]; however, minimally invasive methods have the significant practical advantage for generating iPSCs from patients [22]. We previously reported the generation of T cell-derived iPSCs (TiPSCs), which involves a non-invasive method and requires only small volumes of peripheral blood, which means an increased number of eligible patients [23]. Further to this, the present study sought to clarify whether TiPSCs with genomic T cell receptor (TCR) gene rearrangements could differentiate into functional cardiomyocyte in vitro, by examining the morphology, gene expression pattern, and electrophysiological properties of TiPSC-CMs.

## Materials and Methods

### Cell culturing

The human TiPSC lines, T05 and T07 were generated from human peripheral T cells by introducing *OCT3/4*, *SOX2*, *KLF4*, and *c-MYC* with Sendai virus vectors [23]. The human iPSC line, F16 was generated from human fibroblasts by introducing *OCT3/4*, *SOX2*, *KLF4*, and *c-MYC* with retrovirus vectors [24]. The human ESC line (KhES-2) [25] was obtained from the Department of Development and Differentiation, Institute for Frontier Medical Sciences, Kyoto University and used in conformity with the Guidelines for Derivation and Utilization of Human Embryonic Stem Cells of the Ministry of Education, Culture, Sports, Science, and Technology, Japan. These cell lines were cultured in human iPSC medium consisting of DMEM/F12 medium (Invitrogen) supplemented with 20% Knock-out Serum Replacement (KSR; Invitrogen), 1 mM L-glutamine, 1 mM non-essential amino acids, 0.1 mM β-mercaptoethanol, 50 U penicillin and 50 mg/ml streptomycin (Invitrogen), and 4 ng/ml basic fibroblast growth factor (bFGF; WAKO). The human iPSC medium was changed every other day until the colonies were picked. The iPSCs and ESCs were maintained on irradiated mouse embryonic fibroblast (MEF) feeder cells [26] from wild-type ICR mice in human iPSC medium which was changed every 2–3 days, and the cells were passaged using 1 mg/ml collagenase IV (Invitrogen) every 5–6 days.

### In vitro differentiation of iPSCs and ESCs

The iPSCs and ESCs were harvested using 1 mg/ml collagenase IV, and transferred to ultra-low attachment plates (Corning) in differentiation medium. The differentiation medium consisting of Minimum Essential Medium Alpha Medium (Gibco) supplemented with 2 mM L-glutamine (Invitrogen), 0.1 mM non-essential amino acids (Sigma), 0.1 mM 2-mercaptoethanol, 50 U/ml penicillin and 50 mg/ml streptomycin (Invitrogen), and 20% fetal bovine serum (Gibco). The medium was replaced every second or third day. To promote cardiac differentiation, recombinant human Wnt3a was added to the differentiation medium for the first 4 days of culture at 100 ng/ml [27]. The time window of differentiation for analyzing the beating embryoid bodies (EBs) and cardiomyocytes was 30–60 days from starting the differentiating conditions.

### Reverse transcription-polymerase chain reaction (RT-PCR)

Total RNA was isolated from cells using TRIZOL reagent (Invitrogen) and RNasefree DNase I (Qiagen), according to the manufacturer's instructions. The concentration and purity of the RNA were determined using an ND-1000 spectrophotometer (Nanodrop). The cDNA was synthesized using the Superscript First-Strand Synthesis System (Invitrogen). PCR was performed with rTaq (TAKARA). The primers used for PCR are listed in Table S1.

### Quantitative RT-PCR analysis

Total RNA was isolated and used for reverse transcription with random primers as described by the manufacturer. Real-time quantitative RT-PCR was performed using MX3000P (Stratagene), with SYBR Premix ExTaq (TAKARA). The amount of mRNA was normalized to GAPDH mRNA. Primer sequences and cycling conditions are listed in Table S1.

### Immunofluorescence

The differentiated cardiomyocytes plated on fibronectin-coated dishes were washed once with PBS, and fixed with 4% Paraformaldehyde Fixative (MUTO Pure Chemicals) at 4°C for 30 min. After fixation, cells were treated with 0.4% Triton X-100 in PBS for 15 min at room temperature. After blocked with ImmunoBlock (DS Pharma Biomedical) for 5 min three times, cells were incubated at 4°C overnight with the primary antibodies, followed by washing with the blocking medium and incubation at room temperature for 60 min with the corresponding secondary antibodies. The immunostaining was performed using the following primary antibodies and reagents: anti-α-Actinin (A7811, Sigma-Aldrich), anti-ANP (sc-20158, Santa Cruz), anti- Troponin-I (ab52862, Abcam), anti-GATA4 (sc-1237, Santa Cruz), anti-Nkx2.5 (sc-8697, Santa Cruz), anti-Titin (HPA007042, Sigma-Aldrich), anti-SERCA2 (MAB2636, Millipore), anti-Connexin43(c6219, Sigma-Aldrich), anti-Na/Ca exchanger(MA3-926) and 4′,6-Diamidino-2-Phenylindole (DAPI, Molecular Probes). The secondary antibodies used were anti-rabbit IgG and anti-mouse IgG or IgM conjugated with Alexa Fluor 488 or Alexa Fluor 568 (Molecular Probes). Signal was detected using a conventional fluorescence laser microscope equipped with a colour charge-coupled device camera (BZ-9000, KEYENCE).

### Electron microscopy

Contracting EBs were fixed with 2.5% glutaraldehyde in 60 mM HEPES, pH 7.4 for 120 min at room temperature, and washed three times with 0.2 M phosphate buffer. Cells were post-fixed in 1% OsO_4_ in 60 mM HEPES, pH 7.4 for 120 min at room temperature, and stained en bloc in 2% uranyl acetate for 30 min at room temperature. Tissues were dehydrated with gradually increasing concentrations of ethanol for resin embedding, before thin sectioning and staining.

### Field potential recordings using the on-chip multi-electrode array system

Multi-electrode array (MEA) chips from Multi Channel Systems were coated with fibronectin (Sigma-Aldrich). Beating EBs were plated and incubated at 37°C, and MEA measurements were performed at 37°C, as previously described [7]. The signals were initially processed, and the obtained data were subsequently analyzed with MC Rack (Multi Channel Systems). Data for analysis were extracted from 2–5 min of the obtained data. The recorded extracellular electrograms were used to determine local field potential duration (FPD), defined as the time interval between the initial deflection of the field potential(FP) and the maximum local T wave. FPD measurements were normalized (corrected FPD: cFPD) to the activation rate using Bazett's correction formulae: cFPD = FPD/(RR interval)^1/2^, where RR represents the time interval (in seconds) between two consecutive beats. Isoproterenol hydrochloride (Sigma-Aldrich), E4031 (Sigma-Aldrich), and verapamil hydrochloride (Sigma-Aldrich) were prepared as 1 or 10 mM stock solutions. The FPs were recorded for 5 min, and then drug was added to the medium. After 2–3 min of incubation, the FPs were again measured for 5 min.

### Ca^2+^ Imaging

For calcium imaging, beating EBs were manually dissected and digested with collagenase 4 (Gibco) at 0.1 mg/ml in ADS buffer (116.4 mM NaCl, 5.4 mM KCl, 5.6 mM dextrose, 10.9. mM NaH_2_PO_4_, 405.7 µM MgSO_4_, and 20 mM Hepes, pH 7.3) and plated onto gelatin-coated dishes in differentiation medium. After 7 days in culture the beating cardiomyocyte clusters were loaded with 6 µg/ml Fluo-4 AM (Invitrogen) for 10 min at 37°C. Ca^2+^ transients were recorded in iPSC-CMs using a LSM-710 laser scanning confocal microscope (Carl Zeiss), and images were acquired in the line-scan mode.

## Results

### Characterization of in vitro cardiac differentiation ability of TiPSCs

**Figure 1:**
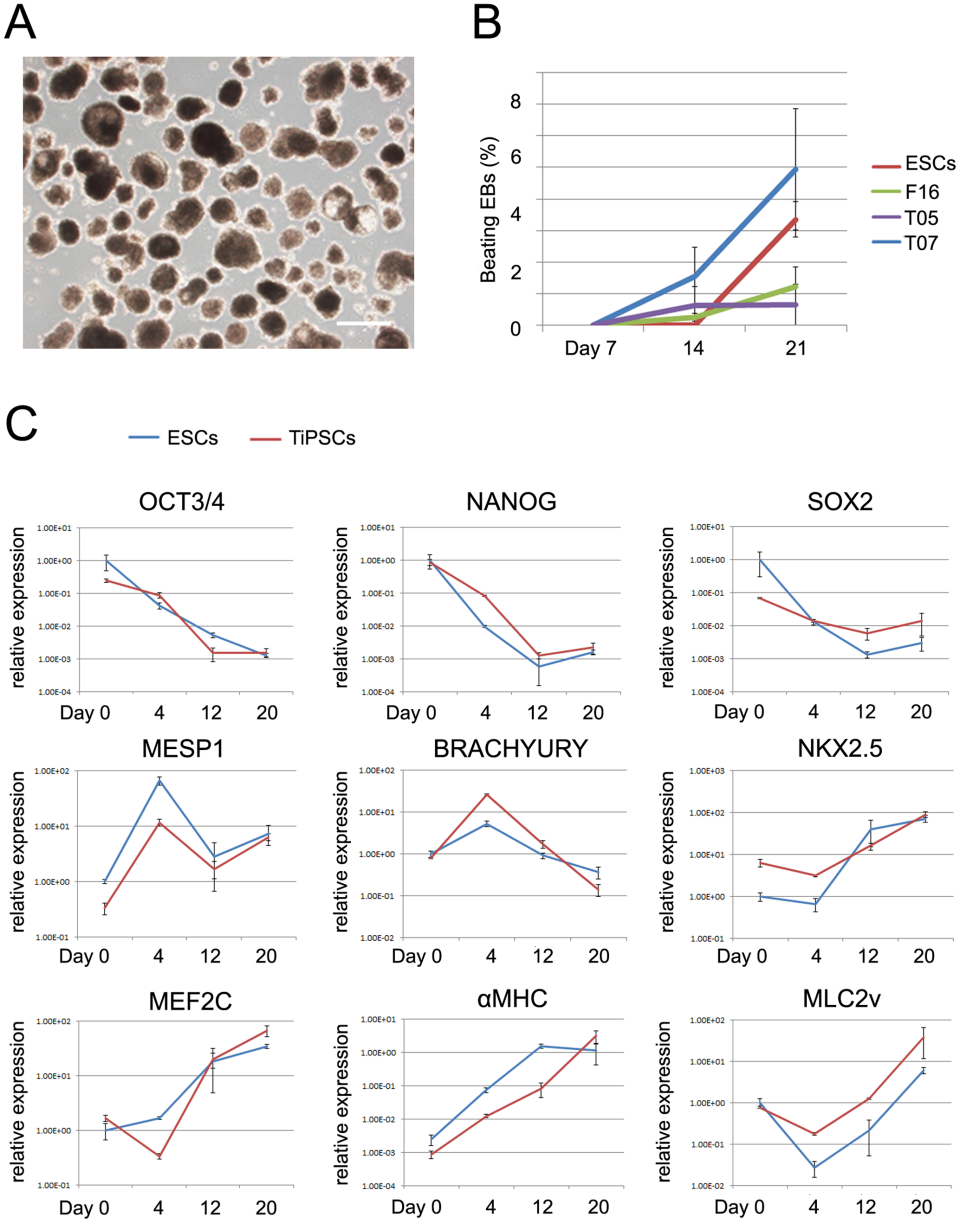
Characterization of in vitro cardiac-differentiation ability of TiPSCs. (A) Morphologies of EBs derived from T07. Scale bar shows 500 µm. (B) Rate of beating EBs derived from ESCs, fibroblast-derived iPSCs line F16, and TiPSC lines T05 and T07 on day 7, day 14, and day 21 of suspension culture. (C) Gene-expression patterns during cardiomyocyte differentiation of TiPSC line T07 and ESCs. Results of QT-PCR studies showed that cardiomyocyte differentiation was characterized by a continuous decrease in the expression of pluripotent markers (*OCT4*, *NANOG*, and *SOX2*) coupled with an initial increase in mesoderm and cardiomesoderm markers (*MESP1* and *Brachyury*). This was followed by the expression of cardiac-related transcription factors (*NKX2.5* and *MEF2C*), and finally by cardiac-specific structural genes (*αMHC* and *MLC2V*). The graph shows an average of three independent examinations. Error bars show means ± s.d.

First, we examined the in vitro cardiac differentiation ability of TiPSCs. We used suspension culture to induce the TiPSCs to form beating EBs, as shown previously for ESCs and iPSCs [28]. The TiPSCs made beating EBs in the same way as ESCs and fibroblast-derived iPSCs (Figure 1A, 1B, Movie S1), confirming that TiPSCs could differentiate into beating cardiomyocytes in suspension culture in vitro.

We also examined the temporal gene expression pattern of derivatives of TiPSCs and ESCs during the in vitro differentiation process. While the expression of undifferentiated pluripotent markers *OCT3/4*, *NANOG*, and *SOX2* decreased similarly, the cardiac-associated transcription factors, *NKX2.5* and *MEF2C*, and the cardiac-specific structural genes, *αMHC* and *MLC2V*, showed high expression in the late stages of floating culture (Figure 1C). On the other hand, primitive streak, mesoderm, and cardiomesoderm markers, *MESP1* and *BRACHYURY*, showed transient expression at the early stage of differentiation (Figure 1C). These results indicated that the expression patterns of undifferentiated and cardiac markers in the derivatives of TiPSCs and ESCs were similar to that observed during embryonic development.

### Molecular and cellular characterization of TiPSC-CMs

**Figure 2:**
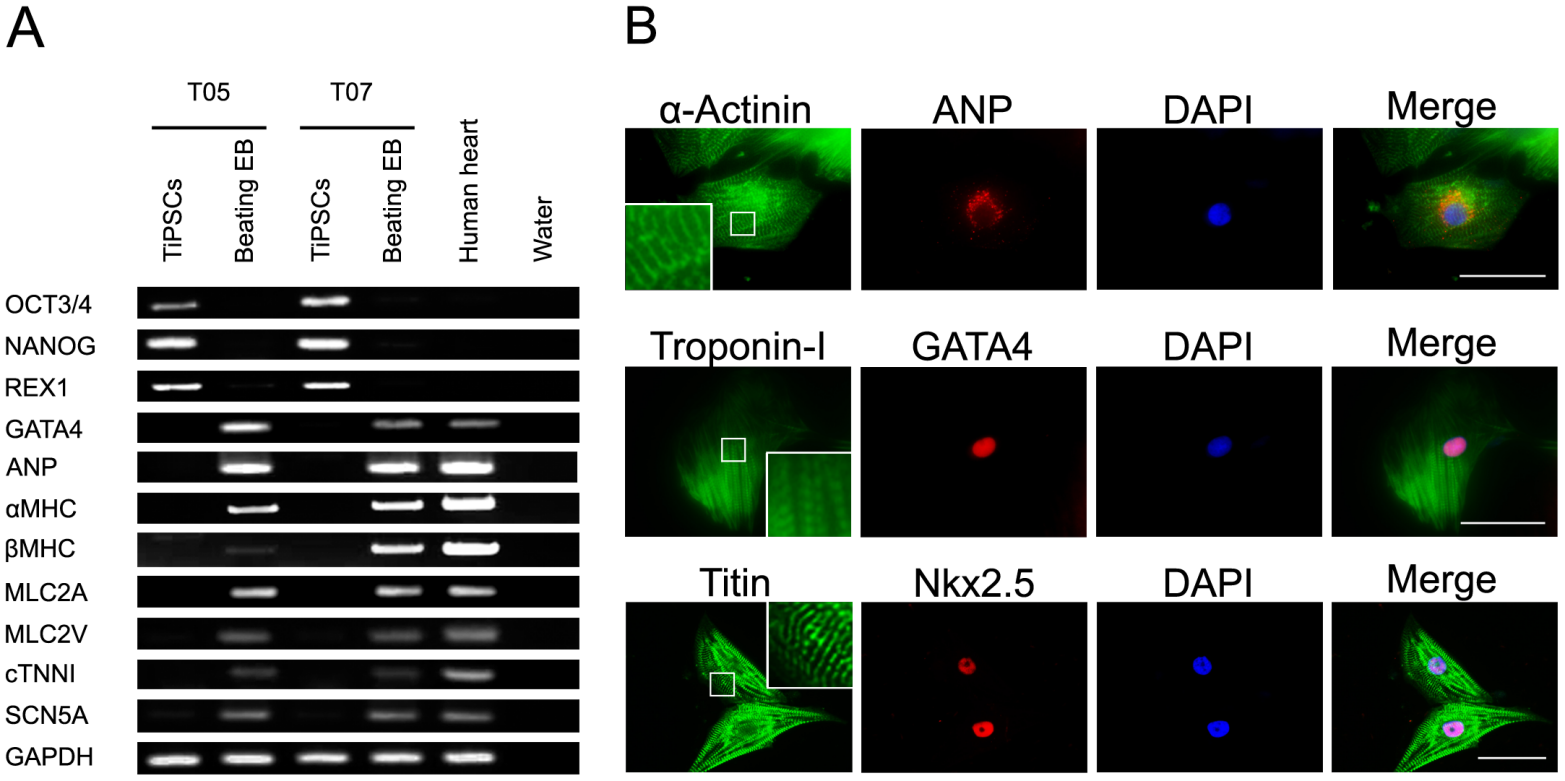
Cardiac-specific markers in TiPSC-CMs. (A) RT-PCR analyses of beating EBs derived from T05 and T07 for the pluripotent marker genes *OCT3/4*, *NANOG*, and *REX1*, the cardiac-related transcription factors *GATA4* and *MEF2C*, and the cardiac-specific marker genes *ANP*, *αMHC*, *βMHC*, *MLC2A*, *MLC2V*, *cTNNI*, and *SCN5A*. (B) Immunofluorescence staining for cardiac-specific markers in cardiomyocytes derived from T07. Scale bar shows 50 µm.

We next examined expression of the cardiac-specific markers of TiPSC-CMs by further analyzing the derived beating EBs. RT-PCR revealed that our TiPSC-CMs properly expressed cardiomyocyte markers and showed decreased pluripotent marker expression (Figure 2A). Immunostaining confirmed the proper expression of cardiomyocyte markers, α-Actinin, Troponin-I, Titin, ANP, GATA4, and Nkx2.5 in the TiPSC-CMs (Figure 2B).

**Figure 3:**
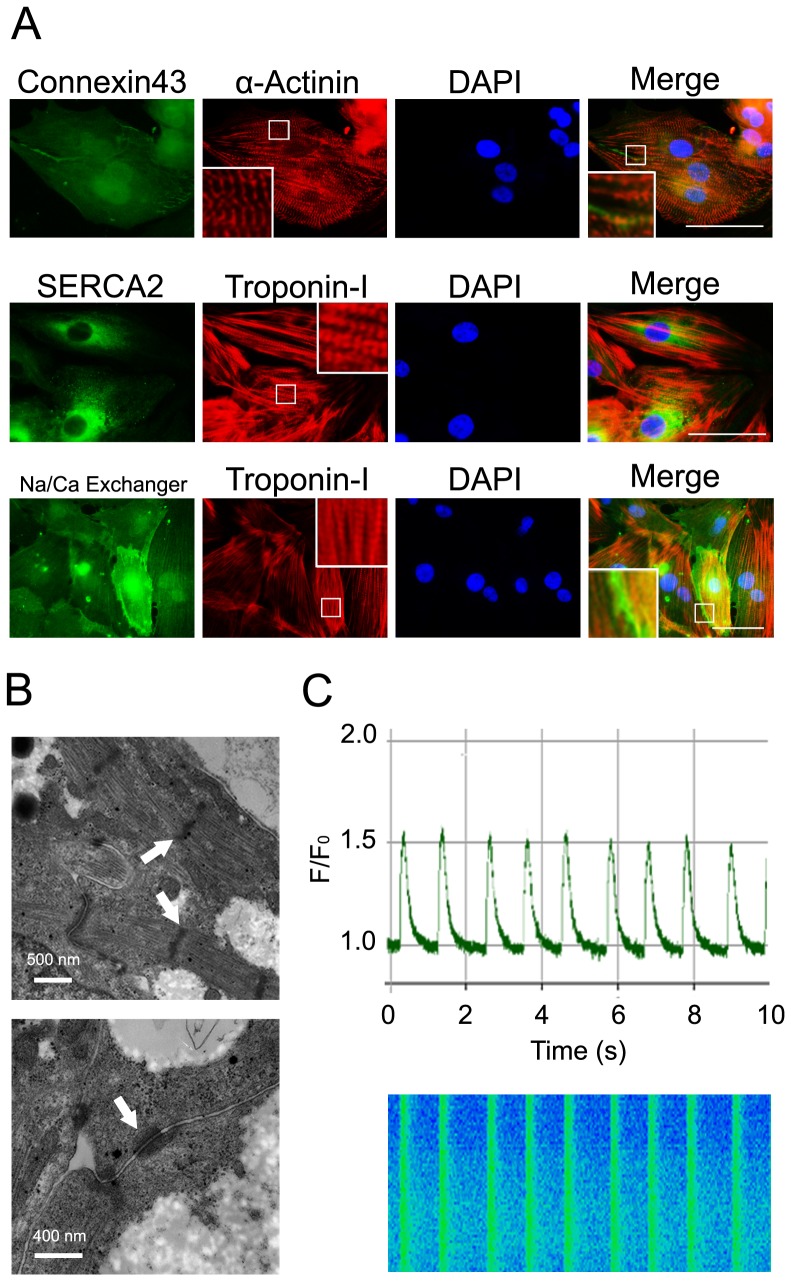
Molecular and cellular characterization of TiPSC-CMs. (A) Immunostaining of proteins involved in cardiac functionality in cardiomyocytes derived from T07. Scale bar shows 50 µm. (B) Electron microscopy analysis of cardiomyocytes derived from T07. Upper micrograph shows sarcomere structures of cardiomyocytes derived from T07. White arrows indicate the Z lines of sarcomere structures. Scale bar shows 500 nm. Lower micrograph shows gap junctions in cardiomyocytes derived from T07 (white arrow). Scale bar shows 400 nm. (C) Line-scan image of Ca^2+^ transient(lower figure) and its average fluorescence intensity in cardiomyocytes derived from T07 (upper graph) were shown. F/F_0_ means fluorescence (F) normalized to baseline fluorescence (F_0_).

We then checked the expression of proteins associated with the functionality of cardiomyocytes other than sarcomeric components in TiPSC-CMs. Connexin43 is the most abundant connexin in the ventricular myocardium; it constitutes gap junctions, which are localized to the cardiomyocyte plasma membrane and electrically couple cardiomyocytes to orchestrate action potential propagation [29]. Immunostaining revealed that Connexin43 to the cardiomyocyte plasma membrane and to the intercellular junctional regions in TiPSC-CMs, suggesting the proper development of gap junctions and intact electric coupling (Figure 3A). The activity of the cardiac sarcoplasmic reticulum Ca^2+^ transport ATPase (SERCA2a) and Na^+^-Ca^2+^ exchanger control cardiomyocyte relaxation and contraction by regulating intracellular Ca^2+^ [30]. In our TiPSC-CMs, SERCA2a was localized to the cytoplasm and Na^+^-Ca^2+^ exchanger was localized to the plasma membrane (Figure 3A). Electron microscopy of the TiPSC-CMs revealed subcellular morphology identical to that observed in native cardiomyocytes such as the sarcomeric organization and gap junctions (Figure 3B). Ca^2+^ oscillations are part of the fully differentiated cardiac phenotype and key to controlled cardiomyocyte relaxation and contraction. To evaluate intracellular Ca^2+^ behavior, Ca^2+^ transients were measured from spontaneously contracting TiPSC-CMs using Fluo-4AM. Line-scan images of Ca^2+^ transient and its average fluorescence intensity in TiPSC-CMs showed Ca^2+^ oscillations (Figure 3C) that indicate electrophysiological function typical of native cardiomyocytes. These findings indicated that the generated TiPSC-CMs were functional in regulating intracellular Ca^2+^.

### Pharmacolobical characterization of TiPSC-CMs

**Figure 4:**
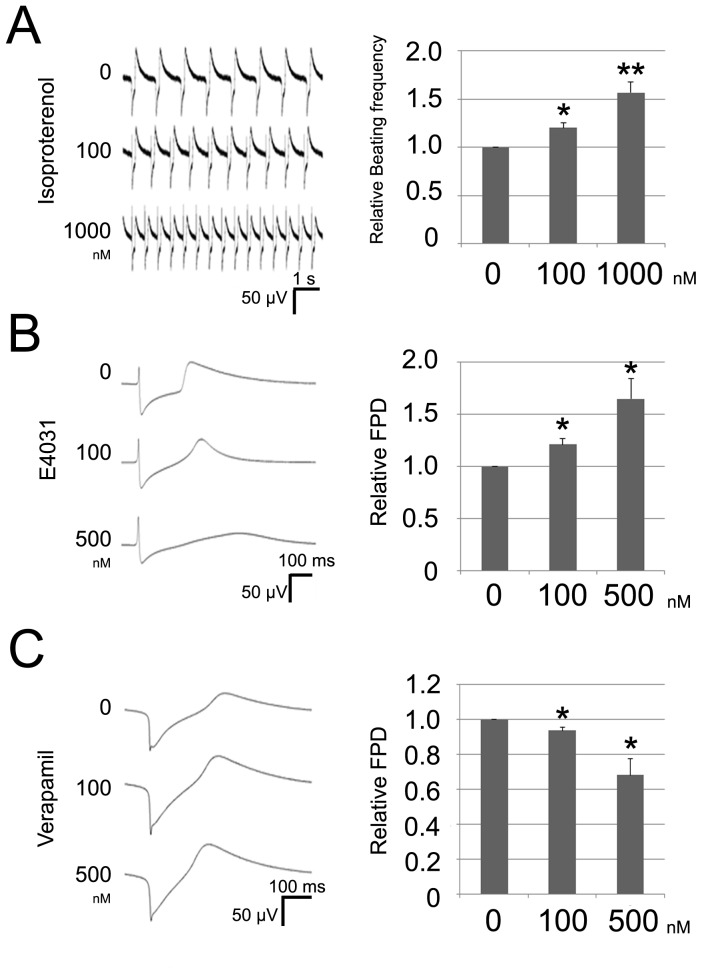
Pharmacological characterization of TiPSC-CMs. (A) Effect of isoproterenol on beating frequency of EBs derived from T07 was shown. The left traces show representative FPs for the various concentrations of isoproterenol. The right graph summarizes the changes in beating frequency by isoproterenol. Error bars show means ± s.d. (B) Effect of E4031 on FP wave form of beating EBs derived from T07 was shown. The left traces show representative FPs for the various concentrations of E4031. The right graph summarizes the changes in FPD by E4031. Error bars show means ± s.d. (C) Effect of verapamil on FP wave form of beating EBs derived from T07 was shown. The left traces show representative FPs for the various concentrations of verapamil. The right graph summarizes the changes in FPD by verapamil. Error bars show means ± s.d. Statistical analyses were performed using the unpaired two-tailed Student's t test (*P<0.05; **P<0.01).

To further evaluate the functionality of TiPSC-CMs, we examined the pharmacological effects of various cardioactive drugs by measuring FPs of the spontaneously beating EBs (Movie S2). To check the response to adrenergic stimulation, we added isoproterenol to the beating EBs derived from TiPSCs, and found a dose-dependent increase in beating frequency, indicating an intact β-adrenergic signaling cascade (Figure 4A). Next, we investigated the effects of ion channel-specific inhibitors on the FP waveform, to determine whether TiPSC-CMs show a native cardiomyocyte-like response to ion channel-specific inhibitors. The IKr blocker E4031 dose-dependently prolonged FPDs in the TiPSCs-CMs (Figure 4B), and the L-type calcium channel blocker, verapamil dose-dependently shortened the length of FPD (Figure 4C). These results indicated that the TiPSC-CMs have identical electrophysiological responses to native cardiomyocytes.

## Discussion

The cardiac differentiation ability of iPSCs and functionality of human iPSCs-derived cardiomyocytes have been characterized in previous reports [6], [7], [31], [32]. However, the only evaluation of functionality in derivatives of human pluripotent stem cells that contain genomic rearrangements in the TCR gene locus was in hepatic cells derived from human TiPSCs [33]. TiPSC differentiation into the three germ-cell layers was shown for in vivo teratoma formation [23], [34]–[37], and mouse mature T cells that contained genomic rearrangements in the TCR gene locus successfully generated monoclonal mice by the nuclear transfer method [38]. However, the functionality of cardiomyocytes differentiated from human TiPSCs in vitro has not been evaluated. In the present study, we demonstrated several characteristics in TiPSC-CMs that are seen in native cardiomyocytes, and also were previously described as cardiac-specific and essential characters in ESC-CMs and iPSC-CMs without genomic rearrangement [6], [39]. Our results thus confirm the potential application of TiPSCs to clinical regenerative therapy for cardiovascular disease and to cardiac disease modeling.

For cardiac cell-based drug screens and cardiac disease research, the minimally invasive methods for tissue sampling involved in generating TiPSCs would be useful for increasing the number of patients who could be subjected to iPSC generation. Our results confirmed the potential of TiPSC-CMs as a cell source of cardiomyocytes with electrophysiological features resembling those of native cardiomyocytes, Therefore, TiPSC-CMs are possible and appealing cell sources for cardiac cell-based drug screens and cardiac disease research.

On another front, TiPSC-CMs have two advantages for transplantation therapy. First, the descendents of TiPSCs that transform into malignant tumors are easily identifiable by their rearrangement patterns. When iPSC-CMs obtained from dermal fibroblasts or other somatic cells are transplanted into diseased patients, there is no good procedure for following the progenies. In animal models, several marker genes can be used to chart the progression and consequences of iPSC-CM transplantation, e.g., GFP and luciferase genes are easily detected and frequently used in experiments with animal models. However, it is not desirable to insert exogenous marker genes into the genomes of human iPSCs for clinical use. In contrast, TiPSCs already have a signature in their specific TCR gene rearrangement, and this could be tracked in TiPSCs descendents [23].

Second, the minimally invasive methods for tissue sampling involved in generating TiPSCs would be especially appealing to allogenic iPSC derivative transplantation because increasing the number of patients subjected to iPSC generation is important for providing HLA-matched cells. Indeed, an HLA-haplotype bank of pluripotent stem cell lines would also be beneficial for reducing the high cost and time spent in generating iPSCs from individual patients [40]. Because TiPSCs can be generated using less invasive tissue sampling compared to dermal fibroblasts by skin biopsy sampling, they are well suited for providing such HLA-matched cell lines for the target population.

In conclusion, TiPSC-CMs have the potential to provide a unique and valuable cell source for clinical regenerative therapy in cardiovascular disease and for cardiac disease modeling.

## Supporting Information

Movie S1 **The beating EB derived from TiPSCs in suspension culture.**
(WMV)

Movie S2 **The beating EB derived from TiPSCs on MEA chips.**
(WMV)

Table S1 **Oligonucleotide primers used for PCR.**
(DOCX)
