# Supplementary material for: Generation and Characterization of Functional Cardiomyocytes Derived from Human T Cell-Derived Induced Pluripotent Stem Cells
Source: PLoS One. 2014 Jan 21;9(1):e85645. doi: 10.1371/journal.pone.0085645 (PMC3897468; doi:10.1371/journal.pone.0085645)
Supplement: Table S1 — Oligonucleotide primers used for PCR. (DOCX) [file pone.0085645.s003.docx]

| **Target genes** | **Primer sequence (forward)** | **Primer sequence (reverse)** | **AT (℃)** | **Number of cycles** |
| --- | --- | --- | --- | --- |
| OCT3/4 | GACAGGGGGAGGGGAGGAGCTAGG | CTTCCCTCCAACCAGTTGCCCCAAAC | 56 | 35 |
| SOX2 | GGGAAATGGGAGGGGTGCAAAAGAGG | TTGCGTGAGTGTGGATGGGATTGGTG | 56 | 35 |
| hNANOG | CAGCCCCGATTCTTCCACCAGTCCC | CGGAAGATTCCCAGTCGGGTTCACC | 56 | 35 |
| REX1 | CAGATCCTAAACAGCTCGCAGAAT | GCGTACGCAAATTAAAGTCCAGA | 56 | 35 |
| MESP1 | CTGCCTGAGGAGCCCAAGT | GCAGTCTGCCAAGGAACCA | 56 | 35 |
| Brachyury | CAACCTCACTGACGGTGAAAAA | ACAAATTCTGGTGTGCCAAAGTT | 56 | 35 |
| MLC2A | CAGGCCCAACGTGGTTCTT | CCATCACGATTCTGGTCGATAC | 56 | 35 |
| MLC2V | CCTTGGGCGAGTGAACGT | GGGTCCGCTCCCTTAAGTTT | 56 | 35 |
| MEF2C | TAACTTCTTTTCACTGTTGTGCTCCTT | GCCGCTTTTGGCAAATGTT | 56 | 35 |
| GATA-4 | TAGACCGTGGGTTTTGCATTG | CATCCAGGTACATGGCAAACAG | 56 | 35 |
| NKX 2.5 | ACCCTGAGTCCCCTGGATTT | TCACTCATTGCACGCTGCAT | 56 | 35 |
| αMHC | TCTCCGACAACGCCTATCAGTAC | GTCACCTATGGCTGCAATGCT | 56 | 35 |
| βMHC | GGCAAGACAGTGACCGTGAAG | CGTAGCGATCCTTGAGGTTGTA | 56 | 35 |
| CTNNI | CCAACTACCGCGCTTATGC | CTCGCTCCAGCTCTTGCTTT | 56 | 35 |
| SCN5A | TGCTGAGTATGCCGACAAG | GTTGATGCACCTCCCAAAT | 56 | 35 |
| GAPDH | CAGAACATCATCCCTGCCTCTAG | TTGAAGTCAGAGGAGACCACCTG | 60 | 35 |

**Table S1: oligonucleotide primers used for PCR**
